# Supplementary material for: Atom probe analysis of electrode materials for Li-ion batteries: challenges and ways forward
Source: J Mater Chem A Mater. 2022 Jan 27;10(9):4926–35. doi: 10.1039/d1ta10050e (PMC8887568; doi:10.1039/d1ta10050e)
Supplement: TA-010-D1TA10050E-s001 [file TA-010-D1TA10050E-s001.pdf]

Electronic Supplementary Material (ESI) for Journal of Materials Chemistry A.
This journal is © The Royal Society of Chemistry 2022

Supporting Information

Atom probe analysis of electrode materials for Li-ion batteries: challenges and ways forward

Se-Ho Kim[a], Stoichko Antonov[a], Xuyang Zhou[a], Leigh T. Stephenson[a], Chanwon Jung[a], Ayman A. El-Zoka[a], Daniel K. Schreiber[b], Michele Conroy[c], Baptiste Gault[a,c,*]

[a]Max-Planck-Institut für Eisenforschung, Düsseldorf, Germany.

[b]Energy and Environment Directorate, Pacific Northwest National Laboratory, P.O. Box 999, Richland, WA 99352, United States

[c]Department of Materials, Royal School of Mines, Imperial College London, London, UK

[*] Corr. Author: b.gault@mpie.de

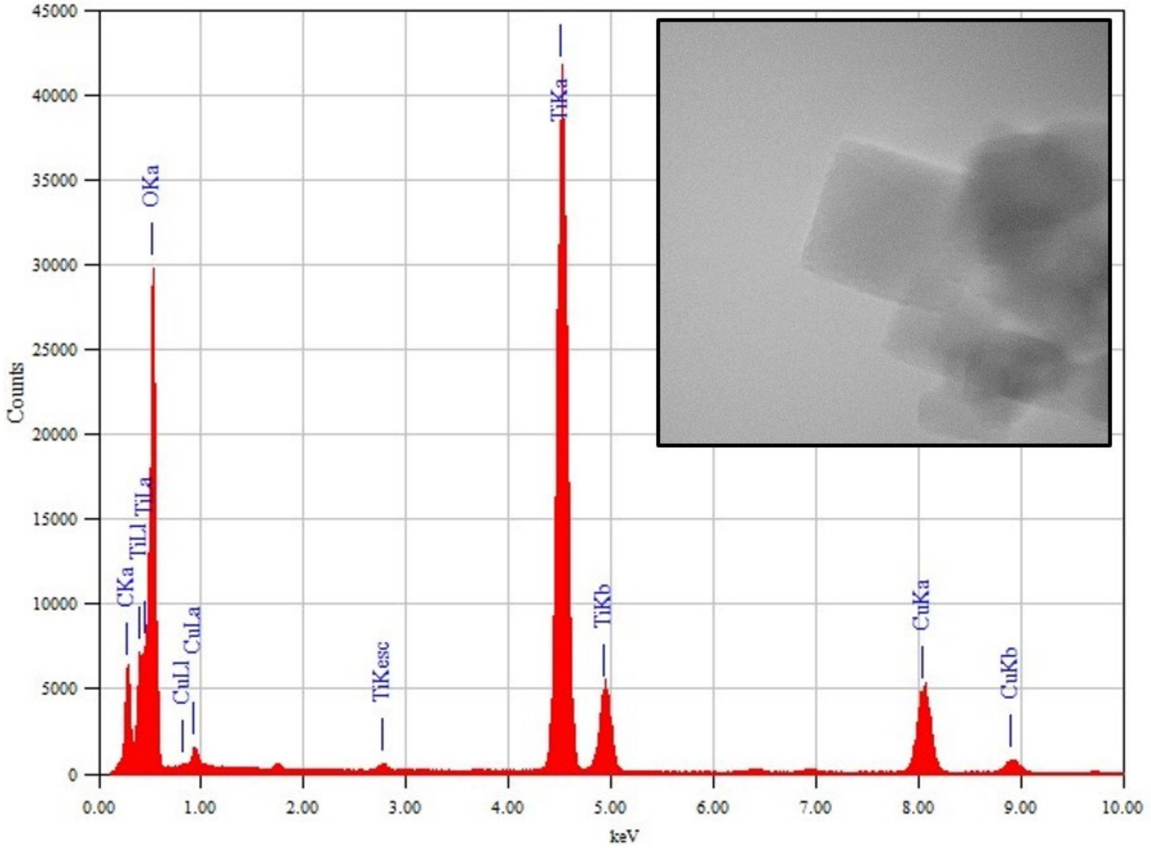

Figure S1. EDX spectrum of as-received $Li_4Ti_5O_{12}$ nanoparticles. Inset image shows the corresponding nanoparticles. Cu and C peaks are originated from a commercial TEM grid. No Li peak detected due to high background signal at low keV range.

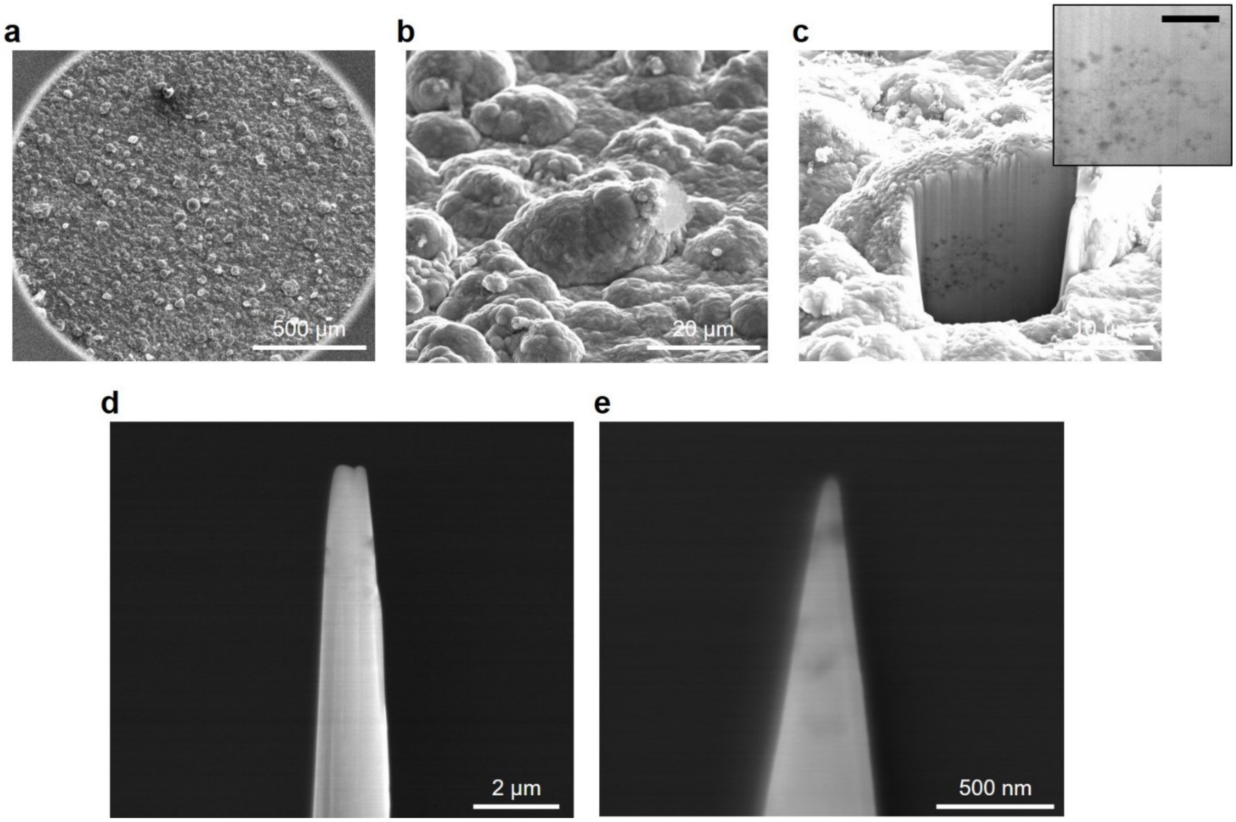

Figure S2. APT specimen preparation from the electrodeposition sample. (a) Surface and (b) side-view images of Ni-electrodeposited $Li_4Ti_5O_{12}$ particles from FIB-SEM. (c) Cross-sectional image after Ga-ion beam milling (30 kV, 21 nA). Inset image shows the embedded particles (scale bar = 2 µm). (d) After the standard lift-out, the sample was mounted on a Si micro-post with Pt/C (30 kV, 89 pA). For the annular milling process, an ion-beam voltage of 30 kV and a current of 0.28 nA were used. (d) A final APT specimen was cleaned by low-ion-beam power (5 kV and 8 pA) for 30 sec.

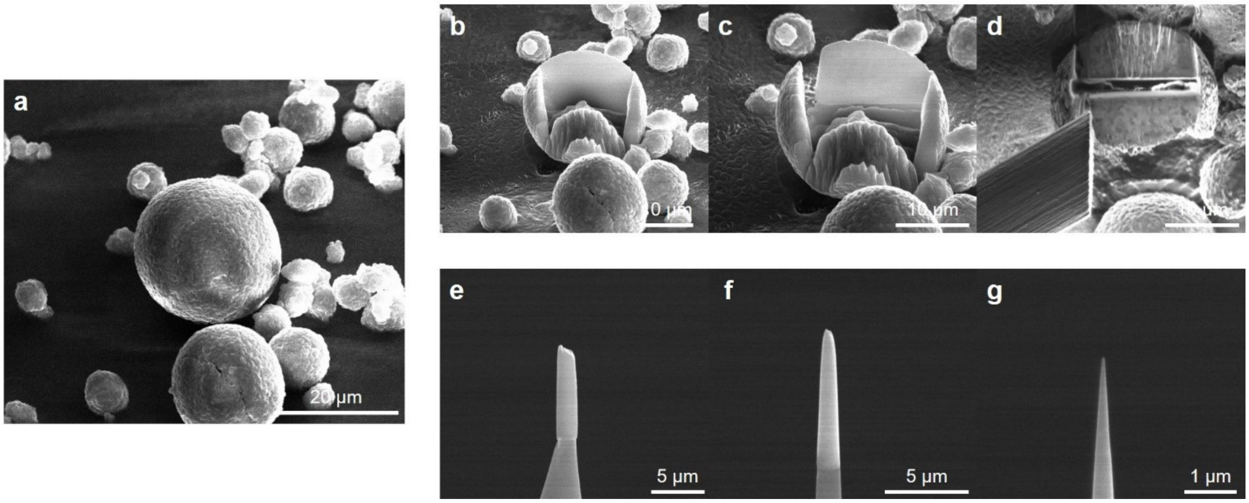

Figure S3. APT specimen preparation from NMC811 particles. (a) SEM-FIB image of as-received NMC811 particles. (b) Trenches from front and back-side Ga millings (30 kV, 21 nA) (c) L-shape cut on the left and bottom side from the lamella. (d) Lift-out process using a micro-omniprobe. (e) Mounted NMC811 sample on a Si post. (f) Annular milling at 30 kV and 0.28 nA. (g) Cleaning process (5kV, 8pA) to get rid of highly damaged regions and/or possible Ga implementation.

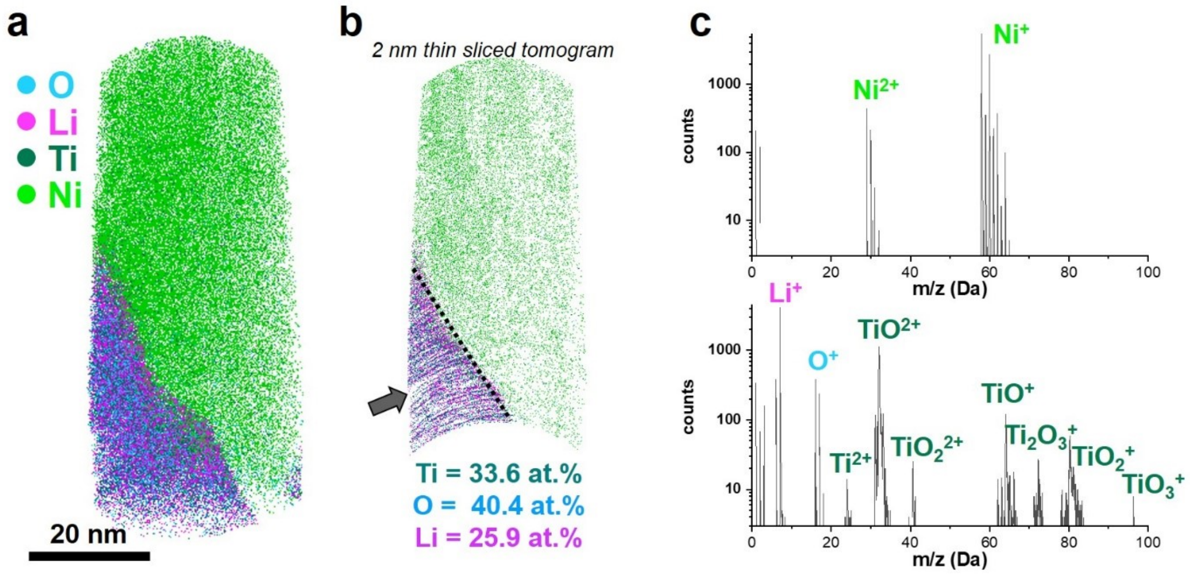

Figure S4. (a) 3D atom map of $Li_4Ti_5O_{12}$ particles in Ni matrix. (b) Tomogram from Figure S3a. Gray arrow indicates that there was micro-facture during the APT measurement. (c) Mass spectra from (top) Ni matrix and (bottom) particle region. Cyan, pink, dark green, and green dots represent reconstructed O, Li, Ti, and Ni atoms, respectively.

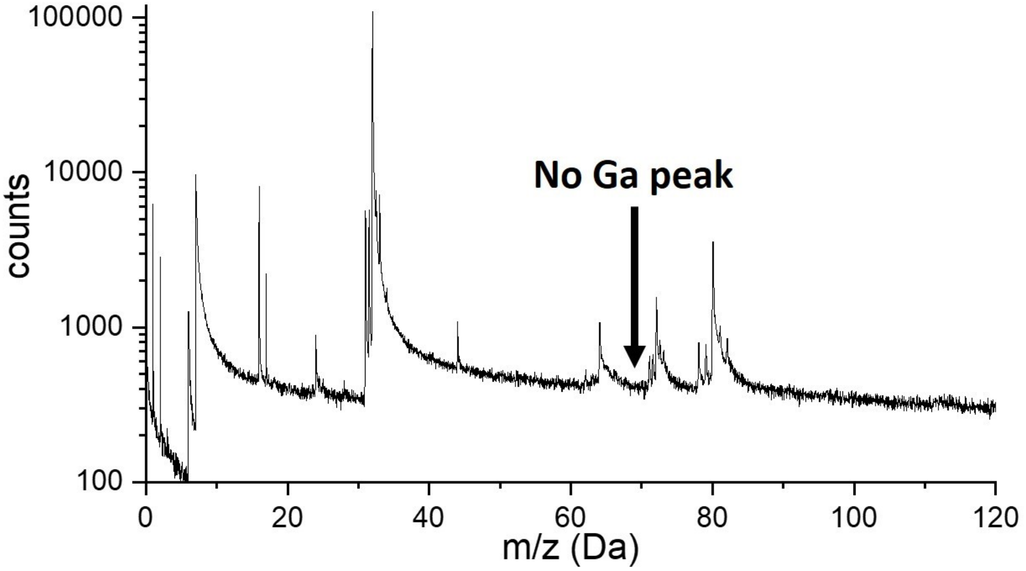

Figure S5. Mass spectrum of LTO bulk acquired from 3000 series atom probe (Figure 1a). Note that no Ga peak at 69 Da detected.

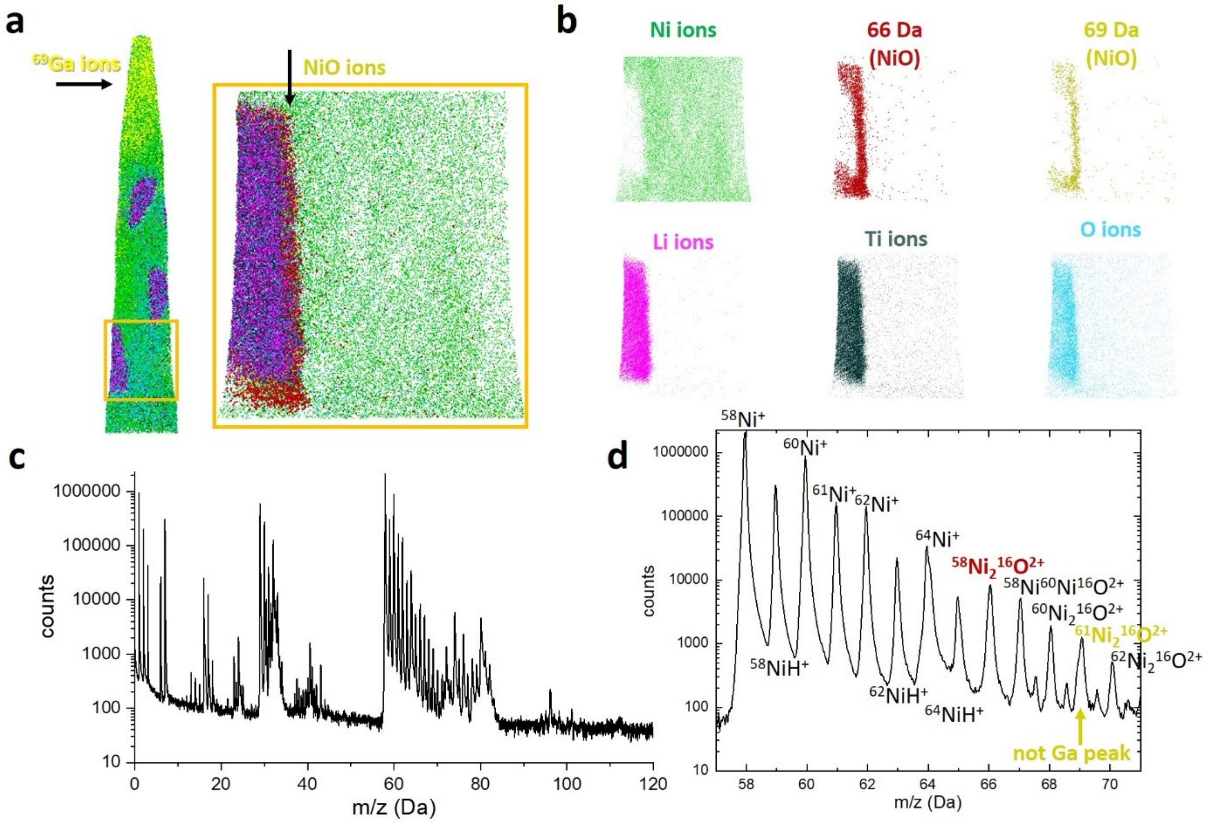

Figure S6. (a) 3D atom map and extracted ROI of LTO-Ni composite (Figure 1d). Note that uncleaned Ga was detected at the beginning of the measurement. (b) Atom map of each ion: Ni, Li, Ti, O, 66 Da and 69 Da ions from the orange ROI. (c) Mass spectrum of extracted region. (d) Selected mass spectrum region between 55-71 Da. The peak at 69 Da was detected first presumed Ga ions however the consecutive peaks at 66, 67, 68, 70 Da and minor peaks at 67.5, 68.5, 69.5 Da implies the 69 Da most likely corresponds to a minor peak of the NiO layer that was developed during the electroplating (Kim et al. Ultramicroscopy 190 30-38).

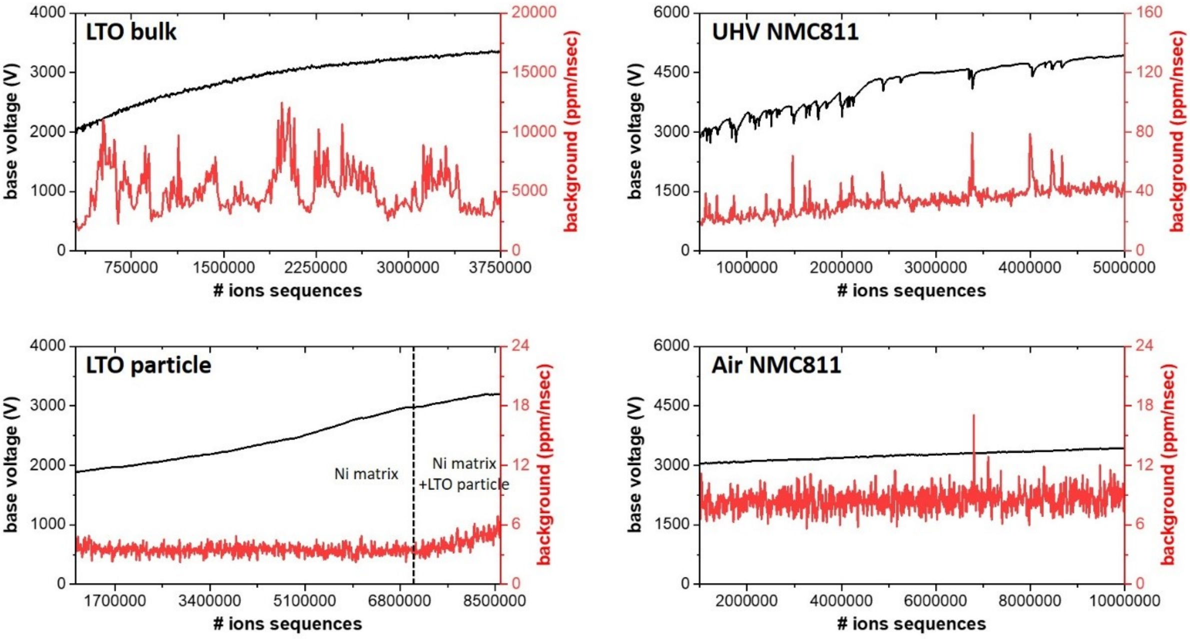

Figure S7. Voltage (black) and noise level (red) curves *vs.* ions sequences on each representative APT measurement of the Li battery materials. Note that the LTO bulk was measured with green-laser assisted 3000 HR. In the LTO particle plot, only Ni matrix ions were detected for first 7M ions until the LTO embedded particle appeared.

For the LTO, since the majority of the data acquired is form the metallic matrix, we also ranged an arbitrary peak at 5 Da (no peak appeared) for both bulk and particle (extracted) LTO datasets at same sampled-ion counts. The LTO bulk dataset had 0.1427 at.% background concentration and for LTO particle, it showed 0.038 at.%. For another an arbitrary peak at 150 Da, the LTO bulk shows 0.6086 at.% and the LTO particle: 0.007 at.%. Overall, this indicates a much higher level of background in the LTO data from the bulk acquired on the LEAP 3000X Si, compared to LEAP 5000 XS.

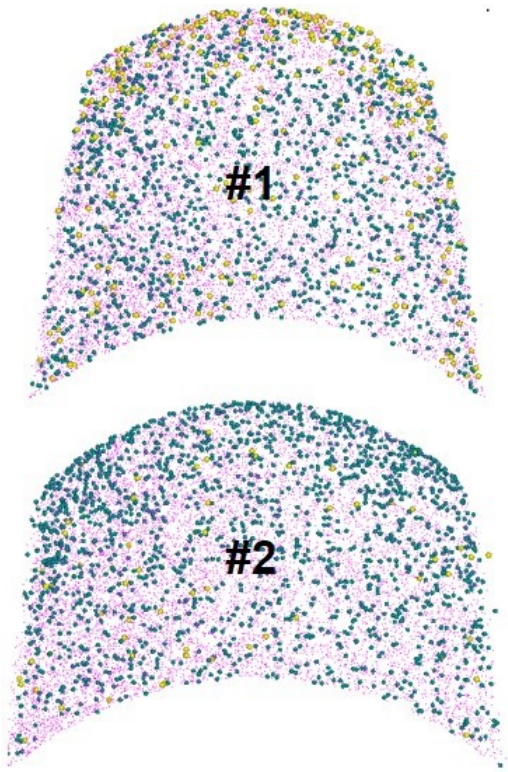

Figure S8. OH_x ions (blue-green) distributions in 2-nm-thin sliced atom map from (up) Figure 3a and (down) Figure 3b. Yellow and pink dots represent the reconstructed Ga and Li atoms, respectively.

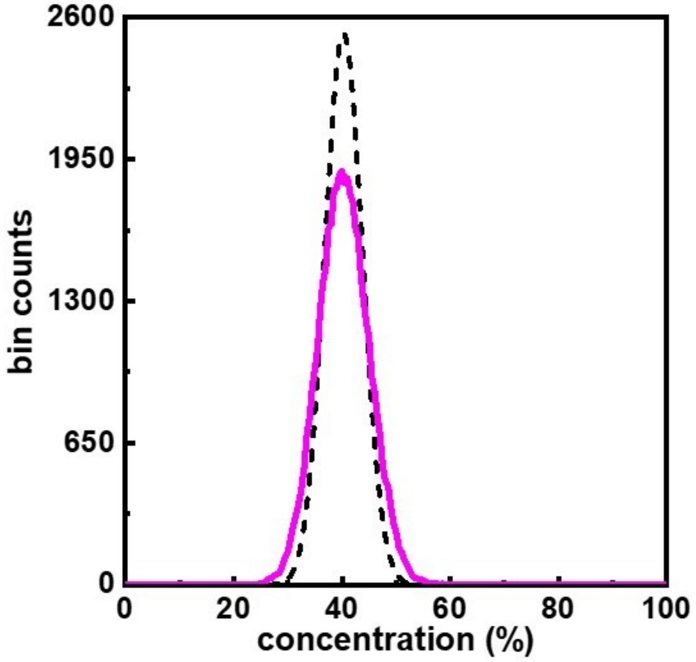

Figure S9. (f) Measured binomial distribution of Li in ion% (pink line) compared to an expected random distribution (dashed line).

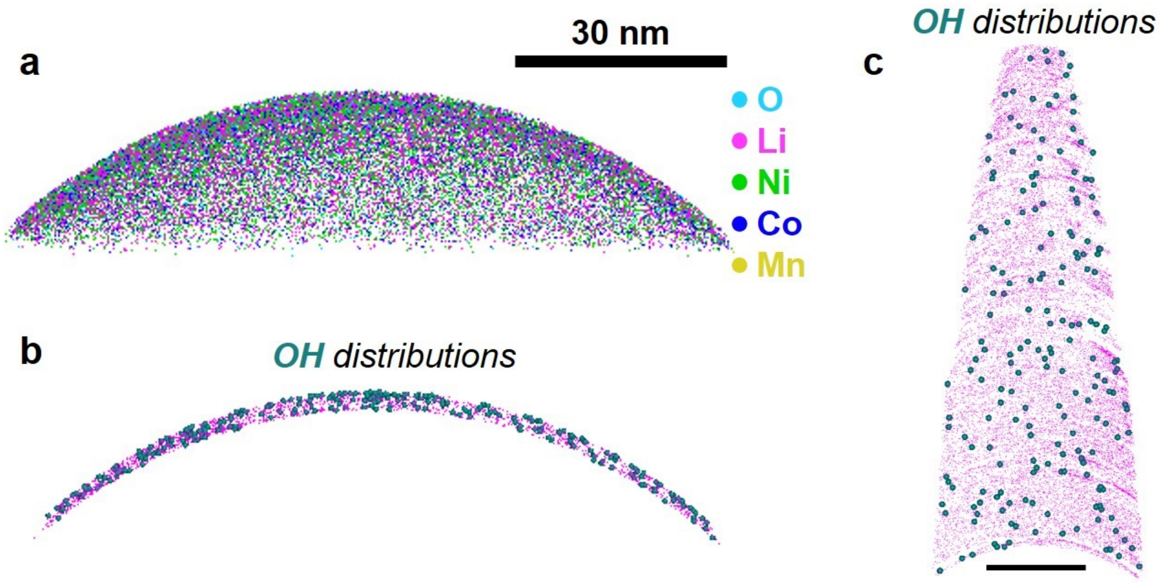

Figure S10. The NMC811 APT result after 24 hr oxidation experiment: (a) 3D atom map and (b) 10nm thin sliced tomogram showing detected OH molecular ions distributions. Cyan, pink, green, blue, and yellow dots represent reconstructed O, Li, Ni, Co, and Mn atoms, respectively. (c) 10nm thin sliced tomogram from the UHV-transferred NMC sample (Figure 2b) showing OH ions distribution.

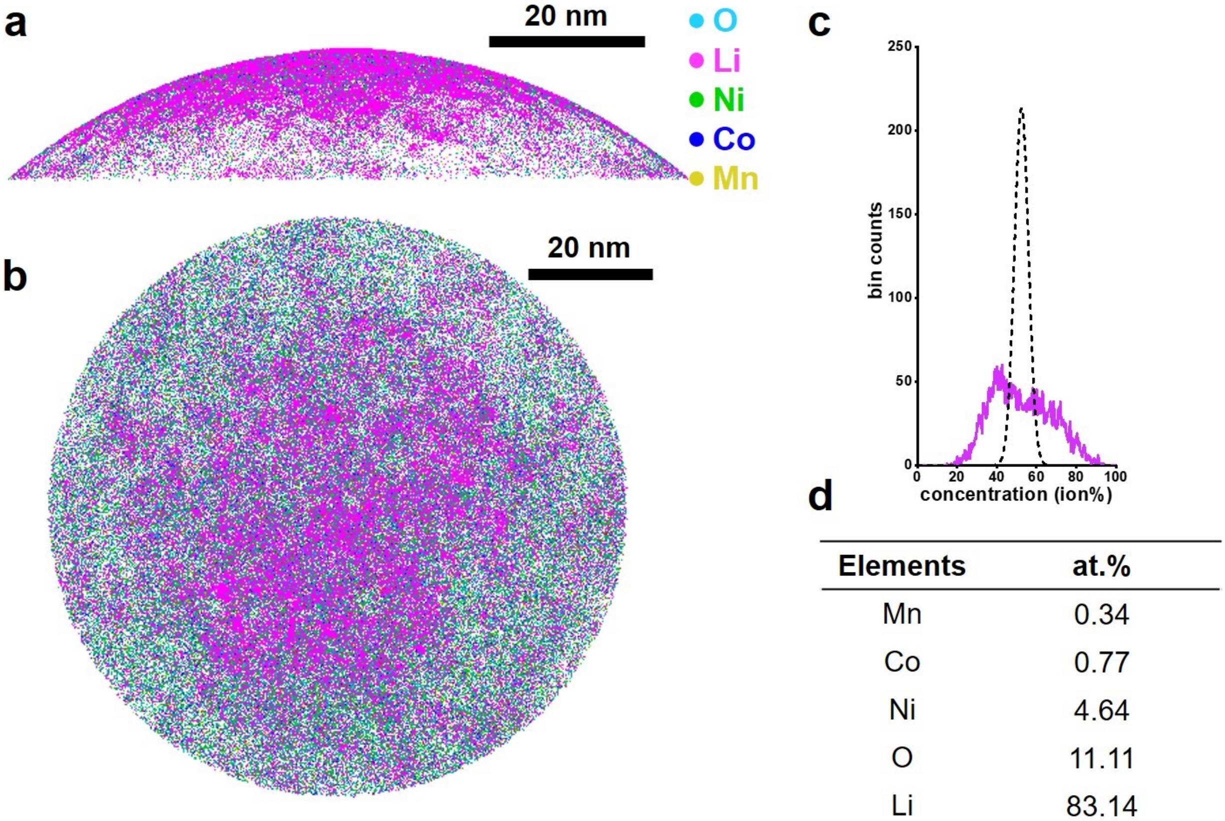

d

Elements	at.%
Mn	0.34
Co	0.77
Ni	4.64
O	11.11
Li	83.14

Figure S11. The air-transferred NMC811 sample measured at high laser energy (20pJ). (a) Side-view and (b) top-view 3D atom maps. (c) Measured (pink) and random (dashed line) binomial distribution of Li. (d) Overall compositional analysis. The measurement was manually stopped.

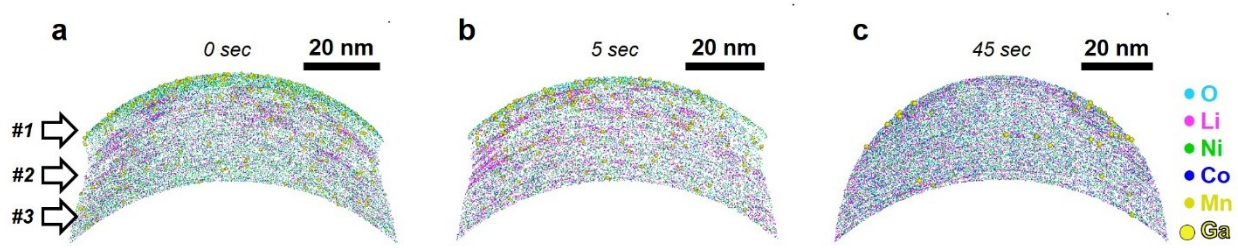

Figure S12. The three NMC811 specimens were prepared using Ga-FIB with different ion beam cleaning conditions to investigate the *(remaining)* Ga effect on the APT measurement. The low Ga ion beam at 5 kV and at 8 pA was used for (a) 0, (b) 5, and (c) 45 sec. All these specimens were transferred in air. Three distinguish regions were detected in the sample (a) and (b). #1 region contains high amount of Ga whereas #2 region showed some Li clusters region. But after 2M ions (region #3), the elements in the NMC811 specimens evaporated homogeneously without any indication of Li hotspots. In the case of the cleaned specimen (c), less Ga ions was detected. The measurement started with region #2 but it got improved to region #3 after <0.5 M ions. All measurement was stopped manually after 5M ions collection.

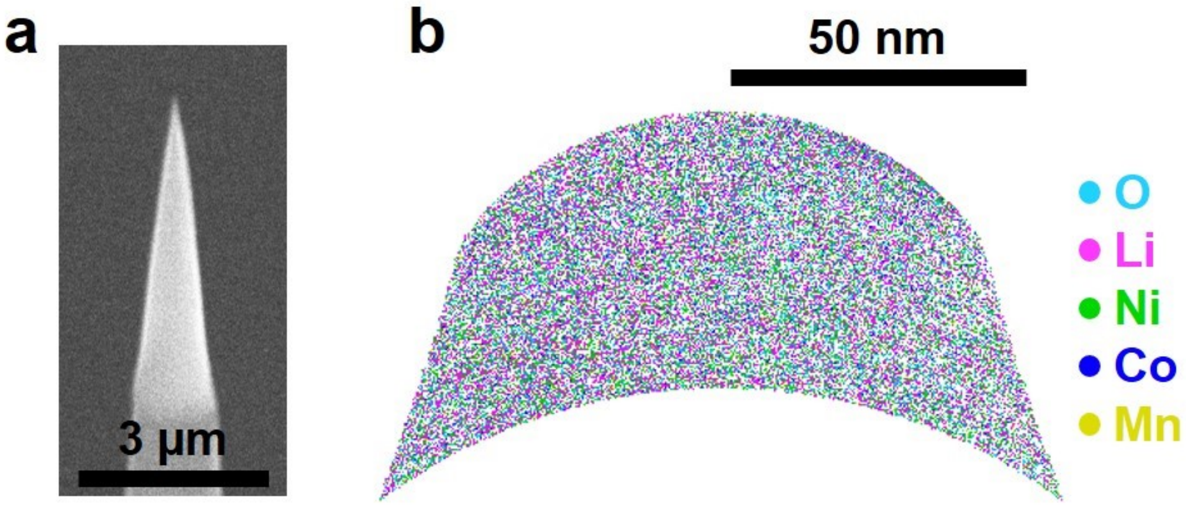

Figure S13. (a) NMC811 specimen fabricated using Xe-plasma FIB. (b) The corresponding APT result.

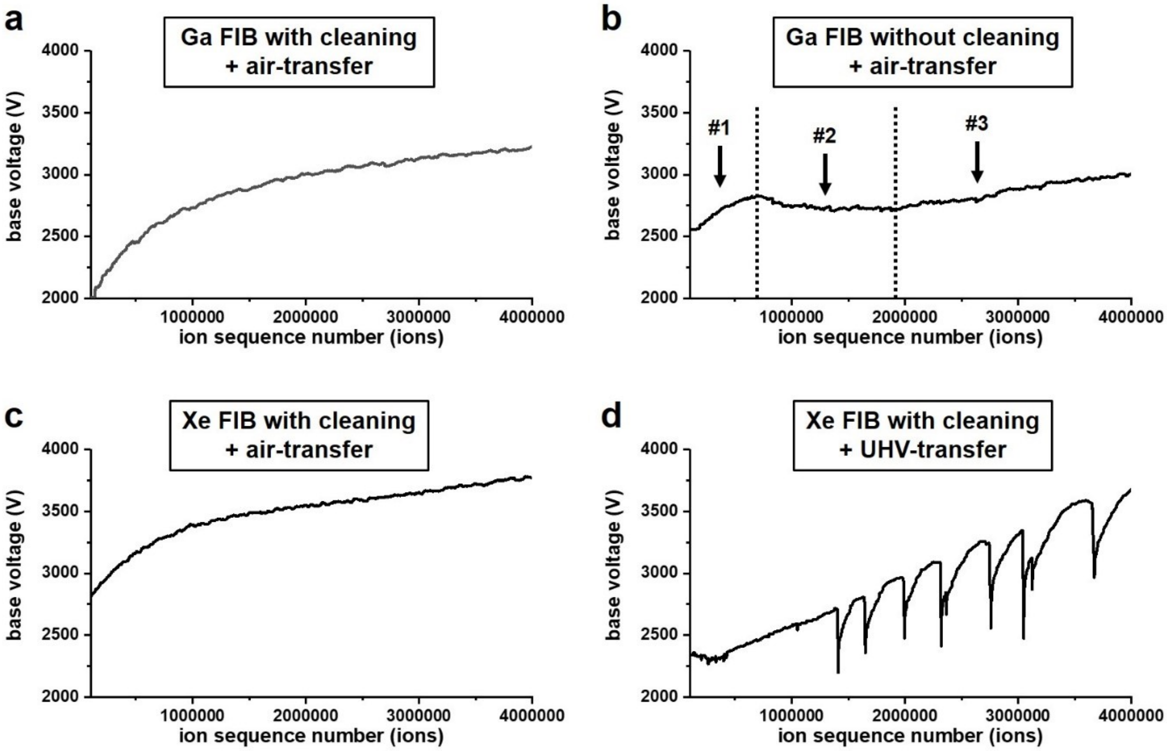

Figure S14. Voltage history curves of the NMC811 specimens fabricated using Ga- and Xe-FIBs: Ga-FIB (a) with and (b) without cleaning process. Xe-FIB (c) with air-transfer and (d) with UHV transfer. Note that there were many micro-fractures after UHV transfer and also note that there were three distinguished regions in the curve (b): Ga-concentrated region, Li-clustered region, and a *pristine* NMC811 region (see Figure S12a). All APT measurement was done at a base temperature of 60 K, a detection rate of 0.5 %, a pulse frequency of 125 kHz, and a laser energy of 5 pJ.

Oxygen flux calculation

The number of oxygen molecules (O_2) impinging on a sharpened specimen at different pressure can be calculated using the classical gas kinetic theory. The flux of O_2 (molecules m^{-2} sec^{-1}) is given by:

$$\Phi = \frac{P}{\sqrt{2\pi M k_B T}}$$

, where P is the gas pressure in Pa; here we assumed all remaining gases are O to yield an upper bound. Another assumption was made that all O gases will be adsorbed on the specimen surface (sticking factor = 1). M, T, and k_B are the molecular weight of the gas in a.m.u., gas temperature in K, and the Boltzmann constant, respectively.

For the case of the (non-cryo) UHV transfer, the overall average pressure of the FIB chamber, intermediate chamber, and the suitcase, was ~10^{-5} Pa. This value yields the flux of 14605 O_2 molecules m^{-2} sec^{-1}. A typical APT specimen has a size of ϕ100 x 100 nm^3. Assuming all O_2 molecules strike only onto a APT specimen, the O_2 adsorption rate will be 4.59 x 10^{-10} molecules sec^{-1} and it requires 69 days to achieve a monolayer coverage of O_2 on the specimen. Unlikely, when the ambient pressure value (e.g. the case of the air transferring) was inputted in the equation, the approximated time for monolayer development of O_2 on the battery specimen is 0.29 sec. This big difference in O_2 monolayer development time suggests that effect of reactive O_2 should be considered.

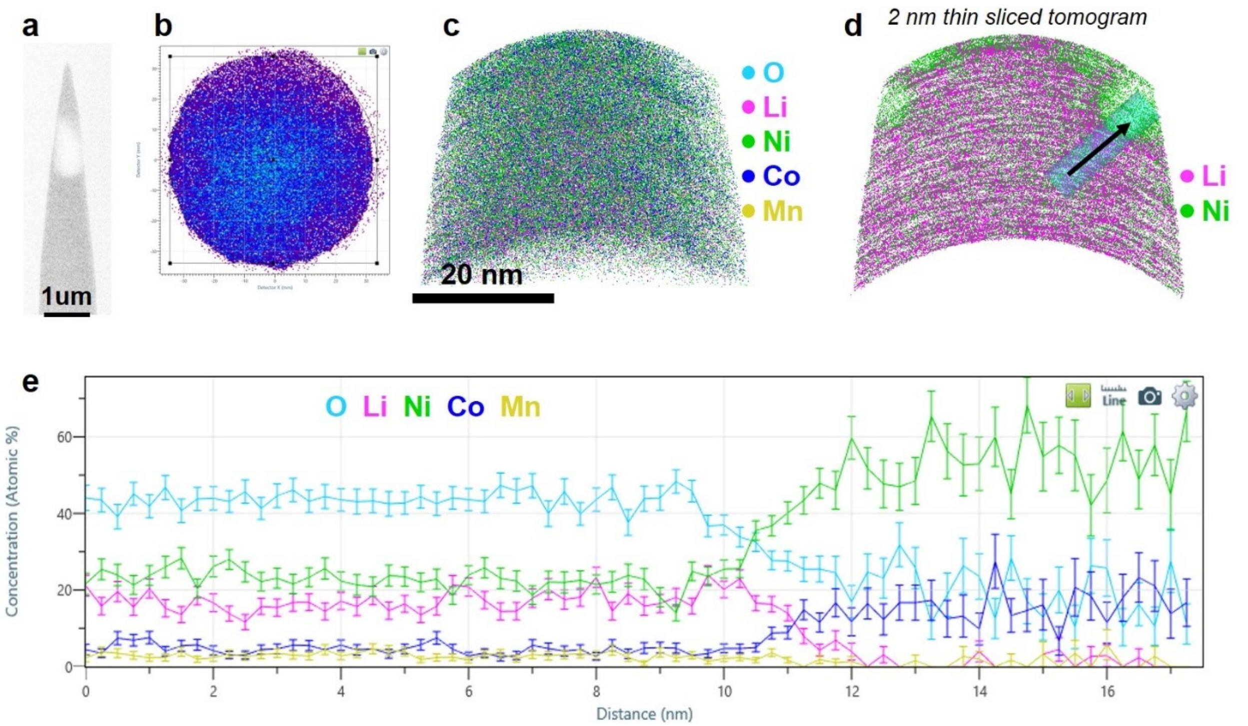

Figure S15. PVD-Ni coated NMC811 APT results with additional Ga milling after deposition: (a) APT specimen after PVD sputtering and Ga-FIB milling. (b) Overall density histogram. (c) 3D atom map of PVD-Ni. Cyan, pink, green, blue, and yellow dots represent reconstructed O, Li, Ni, Co, and Mn atoms, respectively. (d) 2 nm thin sliced along the x-axis. Note that Ni-rich region is detected that is PVD-Ni region. (e) 1D atomic compositional profile along cylindrical region of interest (ϕ5 x 16 nm^3) in Figure S15d. Note that the Co mass-to-charge peak overlaps with the Ni isotope peak; therefore, it is not a Co element in PVD-deposited Ni.

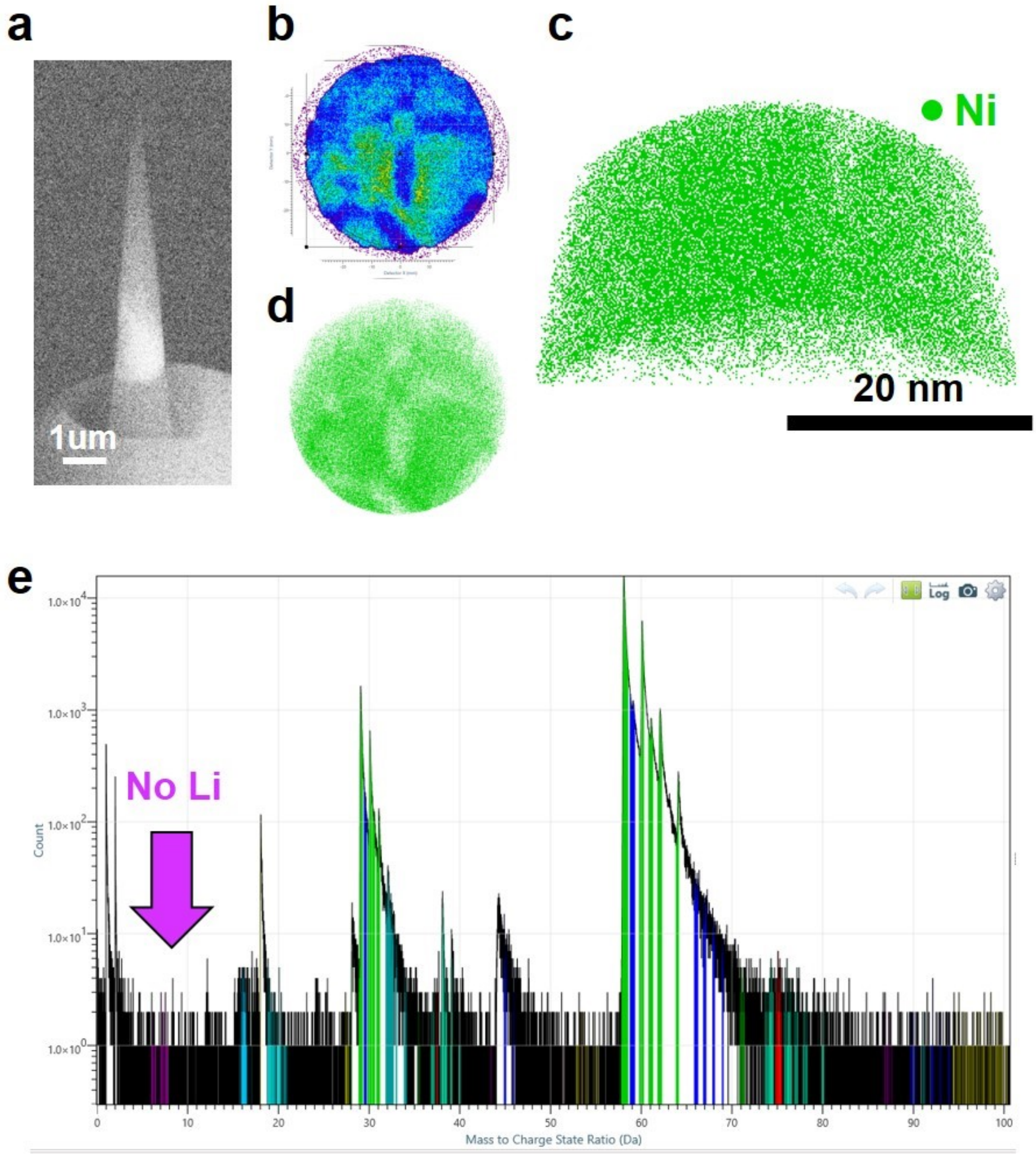

Figure S16. PVD-Ni coated NMC811 APT results: (a) APT specimen after PVD sputtering. (b) Overall density histogram. Note that pole figures are detected. (c) 3D atom map of PVD-Ni. Green dots represent Ni atoms. (d) 5 nm thin sliced along the measurement direction (z-axis). (e) Mass spectrum from acquired dataset. Note that no Li was detected indicating that Li did not electronically diffuse towards to the APT specimen apex.
